# Interposition of the Fracture Fragment of a Vitamin E‐Blended, Highly Crosslinked Polyethylene Liner After Total Hip Arthroplasty: A Case Report

**DOI:** 10.1002/ccr3.9561

**Published:** 2024-11-27

**Authors:** Hiromasa Tanino, Ryo Mitsutake, Hiroshi Ito

**Affiliations:** ^1^ Department of Orthopaedic Surgery Asahikawa Medical University Asahikawa Japan

**Keywords:** blend, interposition, liner fracture, total hip arthroplasty, vitamin E

## Abstract

This is the first case report of a vitamin E‐blended polyethylene liner fracture after total hip arthroplasty. Our case highlights the importance of considering a vitamin E‐blended polyethylene liner fracture, the interposition of fracture fragments between the articulating surfaces after dislocation and blocked successful reduction.

## Graphical abstract

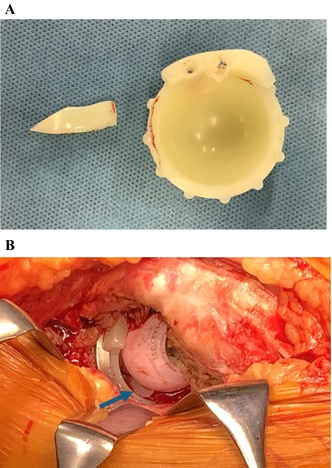

## 1 Introduction

With the introduction of highly crosslinked polyethylene (HXLPE), wear rates and associated periprosthetic osteolysis have been reduced. However, free radicals produced by the crosslinking process, that are not neutralized or stabilized may induce the oxidative degradation of the HXLPE liner, resulting in mechanical failure. The stabilization of HXLPE by thermal treatments after irradiation, such as heat treatments over the melting temperature or annealing below the melting temperature, may decrease the effects of free radicals and promote oxidative stability. The remelting process is a proven method to maximize the quenching of free radicals, but compromises fatigue strength. In contrast, the annealing process maintains polyethylene strength; however, residual free radicals are more effectively eliminated by the remelting process.

Since vitamin E‐stabilized HXLPE (VEPE) has been suggested to prevent mechanical failure caused by oxidative degradation while maintaining mechanical and wear properties, long‐term success and durability of implants after total hip arthroplasty (THA) is expected. Currently available approaches for the manufacture of VEPE are the blending of vitamin E with polyethylene powder prior to irradiation or its diffusion after the crosslinking process [1].

Although a number of case reports have documented the fracture of vitamin E‐diffused HXLPE (diffused VEPE) liners after THA [2, 3, 4, 5, 6, 7, 8], we herein describe for the first time the fracture of a vitamin E‐blended HXLPE (blended VEPE) liner 4 years and 8 months after THA.

## 2 Case History

**FIGURE 1:**
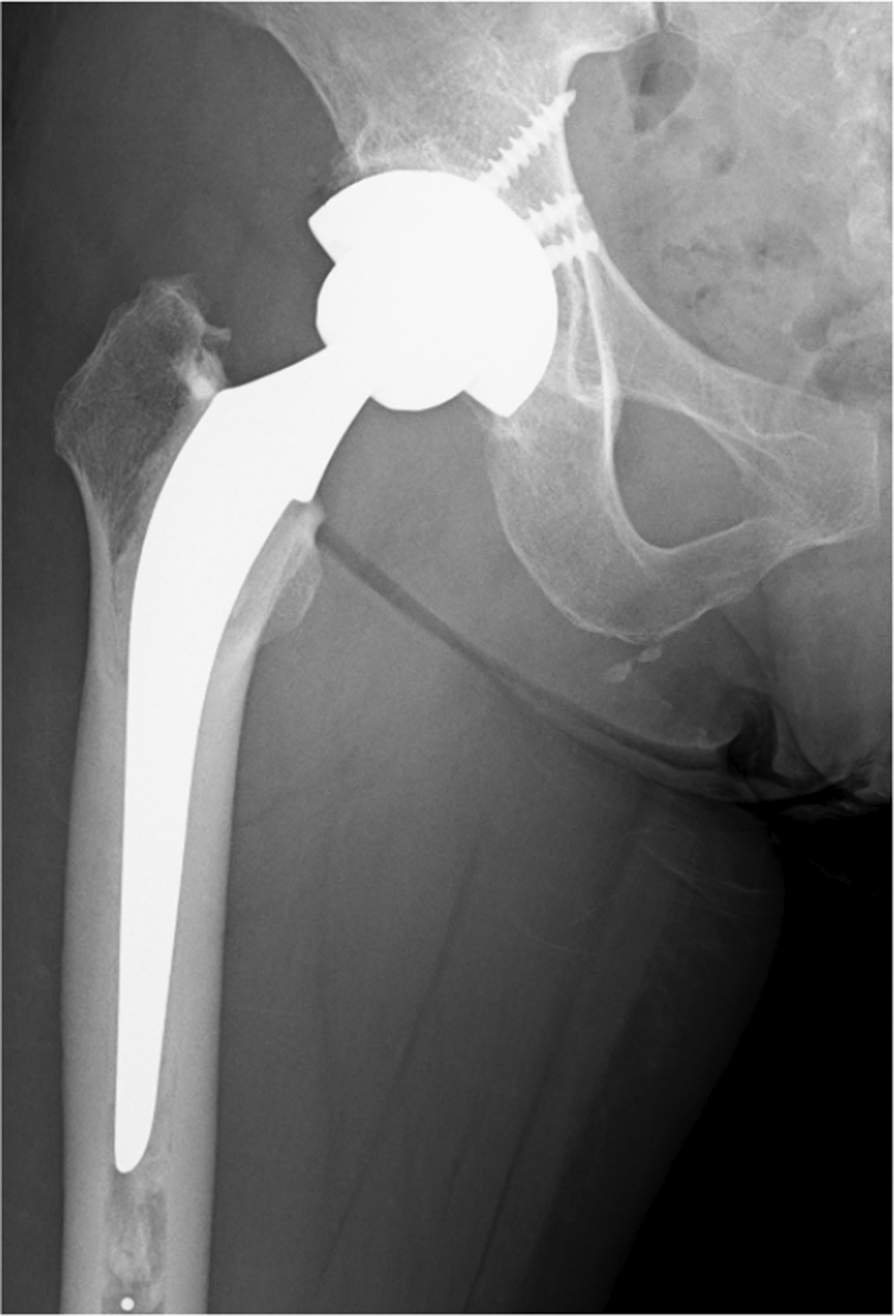
Radiograph after primary THA.

Right THA with a posterior approach was performed for osteonecrosis of the femoral head in a 62‐year‐old woman (body mass index of 28 kg/m^2^) at a local hospital (Figure 1). She received a 50‐mm Continuum cementless cup (Zimmer Biomet, Warsaw, IN, USA) with an elevated rim blended VEPE liner with an inner diameter of 32‐mm (Vivacit‐E; Zimmer Biomet). A cemented CMK Original Concept stem (Zimmer Biomet) size 102 with a +3 mm 32‐mm ceramic femoral head was placed. Surgery proceeded uneventfully. She was a farmer who was able to work and her postoperative course was uneventful. Regular follow‐ups were performed and the appearance of the right hip on radiographs during a visit 4 years after surgery was normal.

**FIGURE 2:**
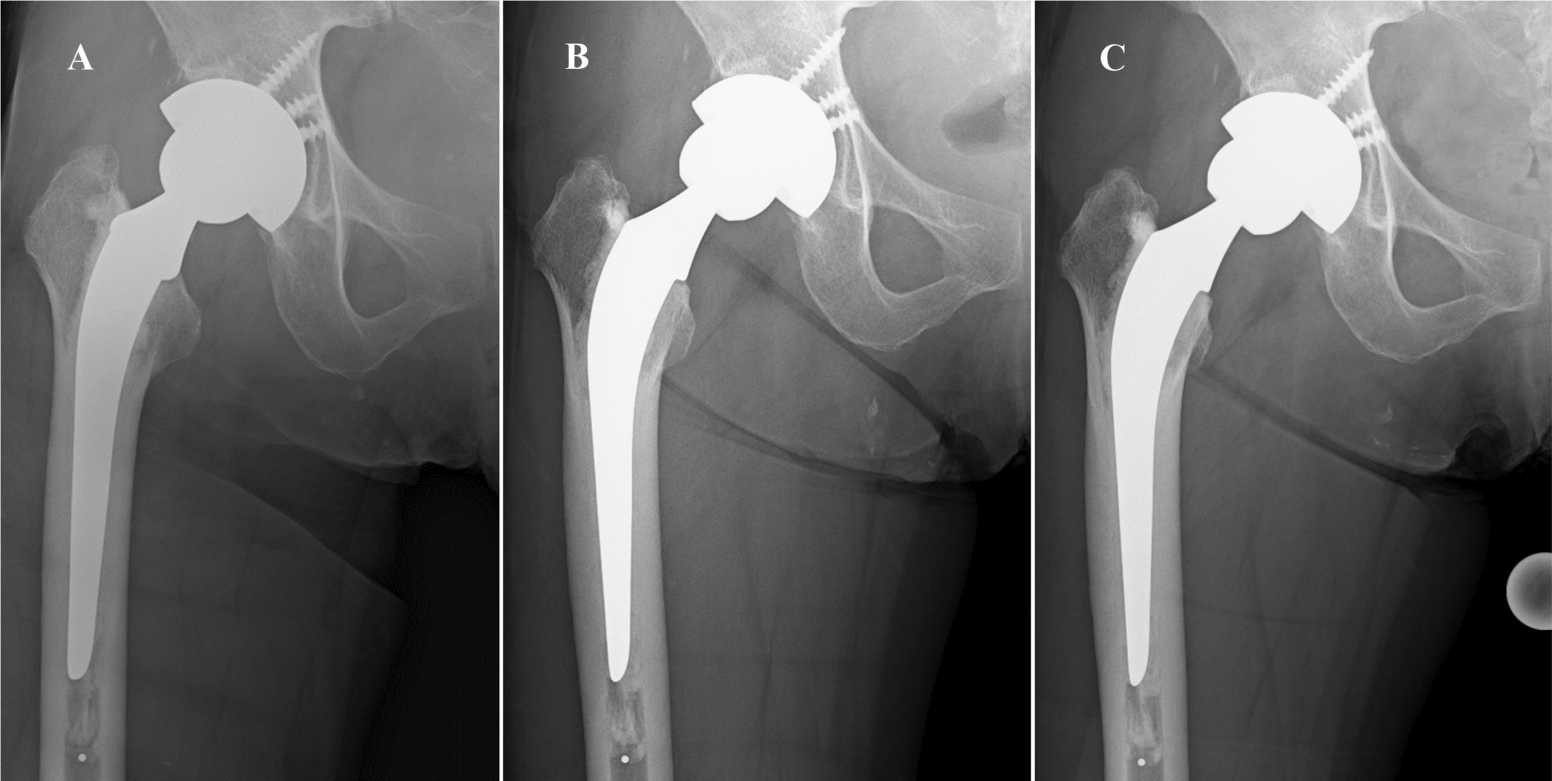
Radiographs after dislocation and closed reduction. Post‐reduction radiographs (A) showed that the femoral head was subtly eccentric compared with a radiograph after primary THA. Radiographs taken during a visit at a local hospital 3 months after dislocation revealed nonconcentric, incomplete reduction (B). In radiographs taken in our hospital, nonconcentric, incomplete reduction was more apparent (C).

Four years and eight months after surgery, she presented to the emergency department of a local hospital because of a fall while working, which resulted in posterior dislocation of the right hip. The hip was reduced at a local hospital and post‐reduction radiographs showed the femoral head was subtly eccentric (Figure 2A), which was not detected until the patient was evaluated at our hospital. After closed reduction, she was able to walk again; however, she complained of groin pain, felt a grinding sensation, and fell several times. Radiographs taken during a visit at a local hospital 3 months after dislocation revealed nonconcentric, incomplete reduction (Figure 2B).

## 3 Methods

She was referred to our hospital for a more detailed examination of groin pain, the grinding sensation, and incomplete reduction. In radiographs taken in our hospital, nonconcentric, incomplete reduction was more apparent (Figure 2C); however, there was no evidence of intra‐articular interposition of the bone or cement between the femoral head and liner in radiographs or on computed tomography scans. The cementless cup and cemented stem were well fixed. Cup abduction was 47° and cup anteversion was 7°. The patient consented for open reduction and revision surgery with or without acetabular cup, liner, and femoral head exchange depending on our intraoperative findings.

**FIGURE 3:**
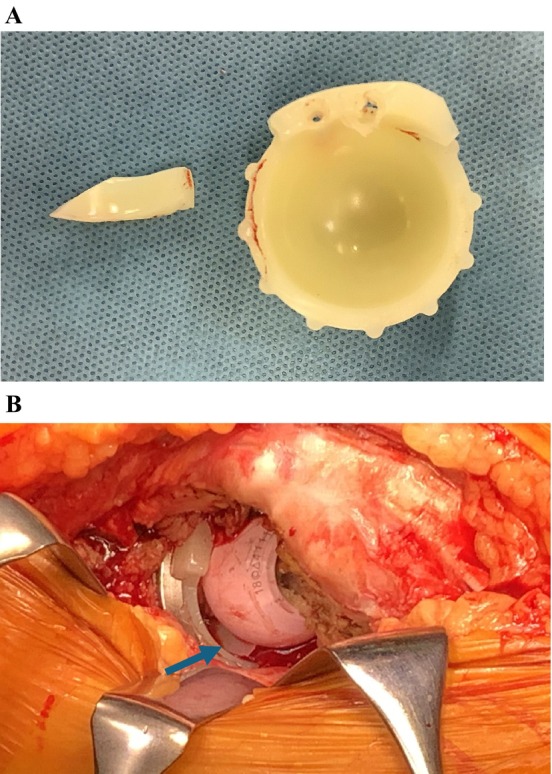
Elevated rim fracture and interposition of a fracture fragment of blended VEPE. The retrieved liner showing a fracture of the posterosuperior elevated rim at the junction of the hemispherical portion of the inner liner and the elevated rim (A). Intraoperative image showing the fracture fragment (arrow) interposed between the femoral head and liner (B) that prevented successful reduction.

Revision surgery was performed 4 months after dislocation through the previous posterior incision. At the time of surgery, the cementless cup and cemented femoral stem were well fixed. Intraoperative findings showed that the liner was fractured at the posterosuperior elevated rim, at the junction of the hemispherical portion of the liner and the elevated rim (Figure 3A). A fracture fragment was detected between the femoral head and liner and had blocked successful reduction (Figure 3B). Excessive polyethylene wear was not observed and, thus, the liner was not subjected to further analyses. Furthermore, there was no evidence of gross infection in the operative field. The acetabular cup was changed to increase cup anteversion using a portable hip navigation system (AR‐HIP; Zimmer Biomet Japan, Tokyo, Japan) [9] because a lack of sufficient cup anteversion may have contributed to dislocation. Stability was increased using a 36‐mm inner diameter, elevated rim blended VEPE liner paired with a 36‐mm cobalt‐chromium femoral head. Revision to the cemented stem was not necessary. Intraoperative stability was confirmed. With new components, hip range of motion was > 90° of flexion with 60° internal rotation and there was no evidence of instability, which was considered to be sufficient [10].

## 4 Conclusions and Results

This is a case of a blended VEPE liner fracture after THA. Interposition of a fracture fragment between the femoral head and liner was detected intraoperatively, and interposition had blocked successful reduction. The postoperative course was uneventful, she felt no pain, and no further dislocations were recorded at 2 months after surgery. Our case highlights the importance of considering a blended VEPE liner fracture and the interposition of fracture fragments between the femoral head and liner as well as avoiding the use of an elevated rim polyethylene liner.

## 5 Discussion

Due to its superior wear characteristics, HXLPE has become the standard polyethylene used in THA. Free radicals produced by the crosslinking process compromise the mechanical properties of the material and may ultimately lead to mechanical failure of the HXLPE liner. HXLPE is stabilized by post‐irradiation thermal treatments, such as remelting or annealing. The remelting process almost entirely eliminates free radicals, but has the undesirable effect of an immediate decrease in fatigue strength. The annealing process reduces the number of free radicals to a lesser extent than that of remelting, but preserves the strength of the material. Therefore, intrinsic limitations persist in terms of the ideal polyethylene properties for both the remelting and annealing processes.

An alternative method that maintains the mechanical and wear properties of HXLPE while quenching/stabilizing any free radicals produced is to incorporate vitamin E, an antioxidant, into HXLPE. Positive short‐ and mid‐term clinical outcomes have been reported for VEPE liners after THA [1]. Currently available approaches include the incorporation of vitamin E into HXLPE by blending or diffusion, each of which had its advantages and disadvantages. In the first approach, prior to its extrusion or compression molding and radiation crosslinking processes, polyethylene powder is blended with vitamin E. Although this achieves a homogeneous distribution of vitamin E, crosslinking efficacy may be decreased. In the second approach, vitamin E is diffused into polyethylene after it is in its near net shape and radiation crosslinking processes. Although the efficacy of crosslinking is maintained, polyethylene may be oxidized prior to the incorporation of vitamin E and an extra homogenization step is needed. Few studies have compared the performance of these methods; therefore, it remains unclear whether the clinical performance of VEPE produced by blending versus diffusion differs [1].

The mechanical properties of VEPE, including wear resistance and fatigue strength after accelerated aging, are excellent and polyethylene‐related complications in VEPE liners are not common; however, fractures around the rims of diffused VEPE liners have been reported after THA. Beecher et al. reported a case of anterosuperior rim fracture 12 months after revision surgery. Since there was no evidence of changes in mechanical material properties in vivo, the fracture was mainly attributed to excessive cup anteversion combined with the use of an offset liner [2]. Bates and Mauerhan also presented a case of the fractured posterorsuperior rim of a liner without a traumatic event 11 months after surgery with a minimum thickness of 4.8 mm [3]. Brazier and Mesko reported a case of superior rim fracture following dislocation 25 months after surgery, and confounders included a traumatic event that caused dislocation, a 36‐mm head combined with a 52‐mm cup, and excessive cup anteversion [4]. Kim et al. also described a case of superior rim fracture with excessive wear 1.5 years after surgery; however, the cause was not identified [5]. Do et al. presented a case of rim fracture 2.5 years after surgery and also was unable to identify the cause [6]. A retrieval study that included four cases of rim fracture in conjunction with vertical cup placement showed that all four liners were offset or were high‐wall liners with a liner thickness of 3.1 or 2.6 mm at the point of fracture [7]. In the Finnish arthroplasty register, there were two cases of diffused VEPE liner fracture in 2723 cases in a mean follow‐up of 5 years [8].

Collectively, the findings of these case reports of diffused VEPE identified the following as risk factors for liner fracture: acetabular component malposition, the use of thin liners with large femoral heads, a history of trauma, and the concentration of stress on the unsupported rim of a liner. However, the cause of fracture was unclear in other case reports. Although several commercially available blended VEPE liners have been used, to the best of our knowledge, we herein present the first case of a blended VEPE liner fracture after THA. In other case reports, diffused VEPE liner fracture occurred within 2.5 years of surgery. In the present case, fracture occurred 4 years and 8 months after surgery. Although the cup was placed inside the Lewinnek safe zone, a traumatic event that included dislocation and/or closed reduction in addition to a high concentration of stress on the elevated rim were regarded as reasons for fracture of the blended VEPE liner. Based on radiographs and intraoperative findings of the present case, elevated rim fracture appeared to have occurred at dislocation or closed reduction, a fragment migrated between the femoral head and liner after several falls, and nonconcentric, incomplete reduction became apparent.

Elevated rim fracture has been reported for HXLPE liners without vitamin E [11] and two cases with the intra‐articular migration of fracture fragments and interposition between the femoral head and liner were reported [12]; however, an intraoperative image of interposition was not provided. We did not suspect an elevated rim fracture or the interposition of fracture fragments before surgery because we had not encountered a case of a blended VEPE liner fracture. Other cup and polyethylene liners were not available at revision surgery. Same design of an elevated rim blended VEPE liner was re‐used in revision surgery. The cup was changed with a portable hip navigation system to increase cup anteversion and a 36‐mm head was used to increase stability. The clinical course of this case will be carefully followed up.

## Author Contributions

**Hiromasa Tanino:** conceptualization, data curation, investigation, methodology, project administration, visualization, writing – original draft. **Ryo Mitsutake:** data curation, investigation, writing – review and editing. **Hiroshi Ito:** data curation, investigation, writing – review and editing.

## Ethics Statement

Institutional Review Board (Asahikawa Medical University Research Ethics Committee) approval was obtained with the reference number AMU‐23188.

## Consent

Written informed consent was obtained from the patient to publish this report in accordance with the journal's patient consent policy.

## Conflicts of Interest

The authors declare no conflicts of interest.

## Data Availability

The datasets generated and/or analyzed during the current study are available from the corresponding author on reasonable request.
